# Supplementary material for: Understanding the combined symptom medication score in the light of contexts and mechanisms
Source: PLoS One. 2025 Jun 16;20(6):e0326143. doi: 10.1371/journal.pone.0326143 (PMC12169515; doi:10.1371/journal.pone.0326143)
Supplement: Attachment — Submitted filename: Response to Reviewers.docx [file pone.0326143.s002.docx]

# Journal requirements:

1.  Please ensure that your manuscript meets PLOS ONE's style requirements, including those for file naming. The PLOS ONE style templates can be found at

[https://journals.plos.org/plosone/s/file?id=wjVg/PLOSOne_formatting_sample_main_body.pdf](https://eur01.safelinks.protection.outlook.com/?url=https%3A%2F%2Ftrack.editorialmanager.com%2FCL0%2Fhttps%3A%252F%252Fjournals.plos.org%252Fplosone%252Fs%252Ffile%253Fid%3DwjVg%252FPLOSOne_formatting_sample_main_body.pdf%2F1%2F010f0194f57f78e7-aa82de54-03ad-43a7-9320-48a0558e695c-000000%2F_wW-y0HNBfrqlbcsZFnULlowhUFsPDRaqkBy8TezQWI%3D197&data=05%7C02%7Canneml%40clin.au.dk%7Cefedeb00666744b259f908dd504d9ebf%7C61fd1d36fecb47cab7d7d0df0370a198%7C1%7C0%7C638755017068949167%7CUnknown%7CTWFpbGZsb3d8eyJFbXB0eU1hcGkiOnRydWUsIlYiOiIwLjAuMDAwMCIsIlAiOiJXaW4zMiIsIkFOIjoiTWFpbCIsIldUIjoyfQ%3D%3D%7C0%7C%7C%7C&sdata=%2BNgAXWlekMFpEasvGrmA8nImJZ9347F%2BGTgGS%2B6j6mk%3D&reserved=0)  and

[https://journals.plos.org/plosone/s/file?id=ba62/PLOSOne_formatting_sample_title_authors_affiliations.pdf](https://eur01.safelinks.protection.outlook.com/?url=https%3A%2F%2Ftrack.editorialmanager.com%2FCL0%2Fhttps%3A%252F%252Fjournals.plos.org%252Fplosone%252Fs%252Ffile%253Fid%3Dba62%252FPLOSOne_formatting_sample_title_authors_affiliations.pdf%2F1%2F010f0194f57f78e7-aa82de54-03ad-43a7-9320-48a0558e695c-000000%2FbGkkpDthZra-KFqrRQTtgvBbbsrWf28S_sr1bVszV0Q%3D197&data=05%7C02%7Canneml%40clin.au.dk%7Cefedeb00666744b259f908dd504d9ebf%7C61fd1d36fecb47cab7d7d0df0370a198%7C1%7C0%7C638755017068963031%7CUnknown%7CTWFpbGZsb3d8eyJFbXB0eU1hcGkiOnRydWUsIlYiOiIwLjAuMDAwMCIsIlAiOiJXaW4zMiIsIkFOIjoiTWFpbCIsIldUIjoyfQ%3D%3D%7C0%7C%7C%7C&sdata=FNDp%2BRZMgvsvRh%2BU%2FFE9H3bGIhwqRGlaXOUweaDQAP8%3D&reserved=0)

**Answer:**

We have thoroughly reviewed the templates to confirm that everything adheres to PLOS ONE’s style requirements.

2. Thank you for stating the following in the Acknowledgments Section of your manuscript:

“The ILIT.NU trial, including this realist evaluation, is financed by Innovation Fund Denmark, Aarhus University, and Aarhus University Hospital.”

We note that you have provided funding information that is not currently declared in your Funding Statement. However, funding information should not appear in the Acknowledgments section or other areas of your manuscript. We will only publish funding information present in the Funding Statement section of the online submission form.

Please remove any funding-related text from the manuscript and let us know how you would like to update your Funding Statement. Currently, your Funding Statement reads as follows:

“The author(s) received no specific funding for this work.”

Please include your amended statements within your cover letter; we will change the online submission form on your behalf.

**Answer:**

Thank you for bringing it to our attention. We have now deleted the Acknowledgement section and updated the Funding Statement.

The data used for this realist evaluation were collected as part of the ILIT.NU trial. The ILIT.NU trial received funding from Innovation Fund Denmark, Aarhus University and Aarhus University Hospital. However, this specific realist evaluation did not receive any funding.

3. In the online submission form, you indicated that [The data that support the findings of this study are available on request from the sponsor, Hans Jürgen Hoffmann. The data are not publicly available due to privacy or ethical restrictions.].

All PLOS journals now require all data underlying the findings described in their manuscript to be freely available to other researchers, either 1. In a public repository, 2. Within the manuscript itself, or 3. Uploaded as supplementary information.

This policy applies to all data except where public deposition would breach compliance with the protocol approved by your research ethics board. If your data cannot be made publicly available for ethical or legal reasons (e.g., public availability would compromise patient privacy), please explain your reasons on resubmission and your exemption request will be escalated for approval.

**Answer:**

The data supporting the findings of this study contain potentially sensitive information, including health-related data and personally identifiable comments from free text boxes that cannot be fully anonymized. As a result, data sharing is subject to ethical and legal restrictions. The authors do not have sole authority over data access decisions.

Our ethical approval requires that data sharing complies with the European Parliament and Council Regulation No. 2016/679 of 27 April 2016 (General Data Protection Regulation, GDPR). Making the data publicly available would conflict with GDPR requirements, which mandate the protection of personal and sensitive information. Therefore, data access is restricted in accordance with these regulations and the guidelines set by The Scientific Ethical Committees for Region Midtjylland, which has imposed these restrictions to protect participant confidentiality.

Requests for access to the data can be submitted to:
**The Scientific Ethical Committees for Region Midtjylland**
Skottenborg 26
DK-8800 Viborg
Tel: +45 7841 0183
Email: [komite@rm.dk](mailto:komite@rm.dk)
Website: [www.komite.rm.dk](http://www.komite.rm.dk)

Reviewer #1:
The method of processing information and monitoring the outcome of treatment through questionnaires in patients with chronic disease is an established method, but your review and analysis are important because of the connection of all the information you have collected.

**Answer:**

Thank you for your positive feedback and for recognizing the significance of this approach. While questionnaire-based outcome monitoring is well established, we appreciate your recognition of our study’s value in integrating qualitative data to provide a comprehensive perspective on the Combined Symptoms Medication Score.

# Reviewer #2:

Dear Author,

This paper presents a very interesting perspective on CSMS and the validity of the questionnaire.

**Answer:**

Thank you.

Could you clarify how you decided on the five preliminary themes? This is not clearly explained in the methods section.

**Answer:**

The five preliminary themes was developed using an inductive approach. This has been elaborated in the methods section to ensure greater transparency.

The results section could be written in a more concise manner. Tables 1 and 4 are somewhat repetitive. Additionally, the refinement of the CMO should be described in the methods section. There are many descriptive statistics that could be presented in a table format.

**Answer:**

We have revised the results section to make it more concise and precise. Additionally, we have moved Table 4 up before Table 3 and incorporated the descriptive statistics into the former Table 4 (now Table 3), removing them from the text to improve readability. You are right that Tables 1 and 4 (now Table 3) are somewhat similar; however, we believe it is important to include both to maintain transparency regarding the differences between the preliminary and final CMOs. We appreciate your insightful suggestions.

In the discussion section, it would be helpful to include a comparison with similar research to better understand the impact of these CMOs on the final study results.

**Answer:**

To compare our findings with similar research, we have addressed this in the discussion section titled *"The Realist Evaluation Approach."* Here, we compare our results with the systematic review by Malengreaux et al. (2022), which examines the use of realist evaluation in health research. This comparison helps contextualize the impact of our identified CMOs and highlights how contextual influences shape outcome measures in realist evaluations.

Overall, I believe this paper could be written in a more engaging way.

**Answer:**

We have carefully revised the manuscript, making it more engaging and your suggestion above about including the descriptive statistics moving it from the text, has improved

Best regards,
Anne Møller Lohmann

Department of Public Health – Respiratory Diseases and Allergy,

Aarhus University and Aarhus University Hospital,

Palle Juul-Jensens Boulevard 99,

8200 Aarhus N, Denmark

Email: anneml@clin.au.dk
