# Supplementary material for: Understanding the combined symptom medication score in the light of contexts and mechanisms
Source: PLoS One. 2025 Jun 16;20(6):e0326143. doi: 10.1371/journal.pone.0326143 (PMC12169515; doi:10.1371/journal.pone.0326143)
Supplement: Attachment — Submitted filename: Response to Reviewer.docx [file pone.0326143.s003.docx]

# Reviewer #2:

Dear Author,

Overall, this is a significant improvement. The revised manuscript offers a valuable contribution to understanding the Combined Symptom Medication Score (CSMS) through a realist evaluation framework.
The manuscript now more clearly explains the inductive approach used for deriving preliminary themes. However, here are some additional comments to improve:

## Answer:

Thank you for your kind and constructive feedback. We’re pleased that the revisions have clarified our approach, and that the contribution is valuable. We address your additional comments below.

1. including a brief example or quote in the methods section illustrating the transition from qualitative data to theme could further support transparency.

## Answer:

Thank you for this helpful suggestion. We have now included a participant quote in the Methods section (Step 1) to illustrate how reflections from the focus groups contributed to the development of a theme. This addition aims to enhance transparency around the transition from qualitative data to theme generation.

2. The comparison with prior realist evaluations (e.g., Malengreaux et al., 2022) is appreciated. The discussion could benefit from a more explicit statement on how this study uniquely contributes to the ongoing refinement of CSMS in clinical research beyond confirming prior concerns.

## Answer:

Thank you for this valuable feedback. We have revised the discussion to more clearly state how the study uniquely contributes to the refinement of the CSMS. Specifically, we now highlight how the findings reveal concrete contextual mechanisms—based on the experiences and reflections of individuals with ARC—that may affect the CSMS’s accuracy and applicability. This aligns with COSMIN recommendations and offers a framework for guiding future validation and refinement efforts.

3. While the study highlights threats to internal validity (e.g., preventive medication use, pollen exposure variability), more direct recommendations for mitigating these issues in future CSMS-based trials would strengthen the conclusion.

## Answer:

We have revised the conclusion to more clearly reflect how future studies may explore ways to address the identified contextual factors that can affect the CSMS—such as variability in pollen exposure, use of preventive medication, foreign travel, and the reporting of unrelated or additional symptoms. We now outline how such findings may inform potential future adjustments to the CSMS. However, we have taken care not to propose direct modifications to the instrument itself, as any such changes would require a new psychometric validation study.

4. Consider also providing more visual clarity in the CMO tables—perhaps bolding key terms or restructuring to facilitate readability.

## Answer:

Thank you for this helpful suggestion. As recommended, we have improved the visual clarity of the CMO tables by bolding the most important elements to facilitate easier reading and navigation of key information.

5. A few sections of the discussion—particularly those addressing additional symptoms—might be more concise for improved flow.

## Answer:

Thank you for the suggestion. We have revised the section on additional symptoms to make it more concise and improve the overall flow of the discussion.

6. If available, additional details on the development or piloting of the updated CSMS (with ad hoc questions) could help contextualize its use and interpretation.

## Answer:

Thank you for the comment. We have added a brief description of how the updated CSMS was developed based on input from focus groups with participants in the ILIT.NU trial. We hope this contextualizes its use and interpretation.

Best regards,
Anne Møller Lohmann

Department of Public Health – Respiratory Diseases and Allergy,

Aarhus University and Aarhus University Hospital,

Palle Juul-Jensens Boulevard 99,

8200 Aarhus N, Denmark

Email: anneml@clin.au.dk
